# “We Need a CERN for AI”: Organized Scientific Interests and Agenda-Setting in European Science, Technology, and Innovation Policy

**DOI:** 10.1007/s11024-024-09568-6

**Published:** 2025-03-17

**Authors:** Anna-Lena Rüland

**Affiliations:** 1 Leiden Institute of Advanced Computer Science, Leiden University, Einsteinweg 55, 2333 CC Leiden, The Netherlands; 2 Global Business School for Health, University College London, UCL East Marshgate, 7 Sidings Steet, Stratford, London, E20 2AE UK

**Keywords:** CERN, AI, STI agenda-setting, Organized scientific interests, European Union, Research policy

## Abstract

How do organized scientific interests shape the agenda of science, technology, and innovation (STI) policy in the European Union (EU)? I address this question by investigating how the so-called “CERN for Artificial Intelligence (AI)” initiative, a proposal to organize Europe’s AI research in a CERN-like ecosystem, made it onto the agenda of EU policymakers. Drawing on the interest group as well as agenda-setting scholarship and triangulating data from multiple sources, I argue that the Confederation of Laboratories for AI Research in Europe, a scientific interest group, pushed the initiative onto the EU’s *informal* (pre-decisional) policy agenda through direct and indirect lobbying. I also demonstrate that CERN for AI has so far failed to enter Brussel’s *formal* (decision) agenda because of turf battles between organized scientific interests; a lack of experience, resources, and time among its advocates; the fragmentation of EU AI research policy and funding; as well as the initiative’s framing. My findings add to the study of STI policymaking in the EU, which has so far focused on individual policymakers, bureaucrats, or political institutions as policy entrepreneurs while neglecting to study the role of organized scientific interests in STI policy agenda-setting.

**Supplementary Information:**

The online version contains supplementary material available at 10.1007/s11024-024-09568-6.

## Introduction

In recent years, artificial intelligence (AI) has caught the attention of policymakers the world over, including in the European Union (EU). The Organization for Economic Cooperation and Development (OECD) defines AI as “a machine-based system that, for explicit or implicit objectives, infers, from the input it receives, how to generate outputs such as predictions, content, recommendations or decisions that can influence physical or virtual environments” (OECD 2024). Among policymakers in Brussels, the regulation of AI has been particularly high on the agenda for the past few years. More recently, however, the so-called “CERN for AI” initiative, a proposal to organize Europe’s AI research in a CERN-like ecosystem, has also made headlines among EU officials (Matthews 2020; Kelly 2021). In this article, I address the question of how CERN for AI made it onto the policy agenda of EU decision-makers. Drawing on the interest group as well as agenda-setting scholarship and triangulating data from multiple sources, I argue that the Confederation of Laboratories for AI Research in Europe (CLAIRE [now known as CAIRNE])^1^, a scientific interest group, pushed it onto the EU’s *informal* (pre-decisional) policy agenda through direct and indirect lobbying. I also demonstrate that CERN for AI has so far failed to enter Brussel’s *formal* (decision) agenda because of turf battles between organized scientific interests; a lack of experience, resources, and time among its advocates; the fragmentation of EU AI research policy and funding; as well as its counter-intuitive framing. In doing so, I add an innovative perspective to the existing scholarship on STI policymaking, which has so far neglected to study the role of organized scientific interests in STI policy. This is surprising given that organized scientific interests have resources and formal structures like offices and spokespersons, which potentially make them more effective in lobbying for specific STI policies than policy entrepreneurs and epistemic communities.

The topic of this article spans three separate literatures, namely those on European science, technology, and innovation (STI) policy; Big Science and Research Infrastructures; as well as on AI governance. Scholarship on European STI policy is less concerned with research infrastructure, but has studied how new research topics emerge. Edler and James (2015), for instance, trace how a new security research theme materialized under the Seventh Framework Program. In doing so, they argue that the European Commission (EC) and mid-ranking officials therein acted as policy entrepreneurs that identified and used a political window of opportunity to push for a new research theme. In the policy enterprise that Edler and James examine, scientific actors seem to have played a subordinate role. Yet Edler and James’s study demonstrates that the STI policy literature is familiar with the role of experts, be they scientific or not, as policy entrepreneurs. What differentiates organized scientific interests from policy entrepreneurs is that the former are professional organizations with resources and formal structures like offices and spokespersons. As such, they might pursue different—and potentially more effective—strategies and tactics than less organized policy actors.

Scholarship on Big Science and Research Infrastructures has extensively studied the role of scientific communities (Hermann et al. 1987; De Mendoza and Vara 2007; Baneke 2020) and individual scientists (Lambright 2002; Panese 2015) in policymaking, particularly around large-scale scientific projects. Most of this literature, however, is under-theorized and historical, focusing on Big Science projects that were established after World War II or during the Cold War. Since then, the political and research landscape has changed considerably, which makes CERN for AI an interesting case to study. By investigating the agenda-setting phase of this particular initiative, it is possible to tease out some, if only anecdotal, differences and commonalities in how scientists bring Big Science projects onto policymakers’ agendas.

Scholarship on AI governance, finally, has overwhelmingly focused on AI governance and ethics more generally (Zhang et al. 2021; Ulnicane 2022; Goos and Savona 2024) as well as the AI Act in particular (Schuett 2023; Novelli et al. 2023). So far, it has neglected to study other aspects of the EU’s AI strategy, including AI research (Mügge 2024: 2), despite the fact that it has repeatedly been argued that “a focus on regulation alone [is] too narrow” (March and Schieferdecker 2023: 11) to effectively shape AI development.

This study adds to all three literatures by providing a theory-guided analysis of how the CERN for AI initiative made it onto the agenda of EU policymakers. It does so in five steps. First, section two presents the heuristic framework that guides the analysis. Thereafter, section three outlines the methods used. Section four details how CERN for AI made it onto the informal policy agenda of EU decision-makers and why it has so far failed to move onto their formal agenda. Considering the existing literature on EU interest groups and large-scale research collaborations, section five discusses the study’s findings as well as their implications for the governance of AI research in Europe. Finally, section six concludes the study by outlining its limitations and future research avenues.

## Organized Interests and Agenda-Setting in the EU

To trace how CERN for AI made it onto the EU policy agenda, I draw on the interest group and EU agenda-setting scholarship, combining them into a heuristic framework that guides the analysis.

### Lobbying Strategies and Tactics

Interest group scholarship has studied the mechanisms through which interest groups manage to put their preferred issues onto the policy agenda or to veto those of others. This type of scholarship defines interest groups as organizations that have members, try to influence public policies, but do not run for office (Dür and Mateo 2016: 2). It largely focuses on business and citizen groups as well as their lobbying strategies. Associations that champion the interests of specific professions, such as scientists, are rarely studied in-depth. When interest group scholars do investigate professional associations, it is assumed that they have more in common with citizen than with business groups (Dür and Mateo 2013: 673).

All interest groups lobby through direct and indirect strategies (Berry 1977). In the pertinent scholarship, strategies are understood to be “overall approaches to seeking influence” (Binderkrantz and Krøyer 2012: 124). They are distinct from tactics in that the latter are “specific activities [that groups] engage in” (Binderkrantz and Krøyer 2012: 124), or to put it differently, tactics are the empirical manifestations of lobbying strategies. Using direct lobbying strategies requires interest groups to approach public decision-makers, for example through tactics like contacting and arranging meetings with members of parliament. Interest groups—scientific ones in particular—are valued for and granted access to these decision-makers because of their expertise (Eising 2007: 352). In other cases, interest groups may want to target the media, other groups that have a stake in the issue at hand, or the public. To do so, they rely on indirect lobbying strategies and tactics like giving interviews or publishing open letters (see Table 1 for an overview).

Interest groups lobby during all five phases of the policy cycle (agenda-setting, policy formulation, adoption, implementation, and evaluation). The agenda-setting phase, however, is particularly important for them for two reasons. First, it is during this phase that policy issues are raised and defined to get the attention of policymakers (Peters 1994: 9), which is a crucial “precondition for policymaking” (Princen 2011: 927). Second, policymakers are particularly responsive to input during this phase because it is the most uncertain one in the policy cycle, which increases the need for expert advice (Stevens 2023: 6). It is here that the interest group and agenda-setting scholarship intersect.

**Table 1:** Overview of direct and indirect lobbying strategies and tactics; own illustration based on Binderkrantz (2005)

Direct strategies		Indirect strategies	
Administrative	Parliamentary	Media	Mobilization
Contacting public servants	Contacting parliamentary committees	Giving interviews	Arranging public meetings
Responding to requests for comments	Contacting members of parliament	Issuing press releases	Publishing open letters
Using public consultations		Writing opinion pieces	Publishing petitions

### Agenda-Setting Processes and Tools in the EU

The scholarship on agenda-setting often characterizes the EU as a “prospective agenda-setter’s paradise” because there are a number of access points for actors trying to gain influence, including organized interests (Peters 1994: 21). This institutional openness, however, does not automatically guarantee that an issue which is being considered (*informal* agenda) will also be acted upon (*formal* agenda) (Princen 2016: 351). For an issue to be put on the formal agenda, it typically has to go through four “career” stages (Cobb et al. 1976: 1976). These four stages are issue initiation, issue specification, issue expansion, and issue entrance. Issue initiation “relates to the way an issue is created,” while issue specification refers to the translation of an issue into specific demands (Princen and Rhinard 2006: 1121). Issue expansion, in turn, “concerns the way issues are moved beyond the initial actors to a wider set of participants” and issue entrance occurs when an issue is placed on the agenda of decision-makers, typically to be acted upon (Princen and Rhinard 2006: 1122). While these issue stages are ideal-typical and hence unlikely to accurately reflect the empirical reality, they help to break down a complex process into “more manageable components” (Stevens 2023: 3).

The openness of the EU institutional ecosystem poses two challenges for prospective agenda-setters (Princen 2011). First, they need to attract attention to an issue. Second, they have to credibly demonstrate that the EU is the right venue to address an issue (Princen 2011: 928). To tackle these two challenges, framing is key. In interest group scholarship, framing is generally defined as a “spinning strategy” that organized interests use to “shape the debate surrounding a policy issue with the aim of influencing policy outcomes” (Voltolini and Eising 2017: 355). Interest groups can, for example, attract attention to an issue among EU policymakers through “big words” or “small steps” (Princen 2011: 933). Framing through big words requires interest groups to connect an issue to values that are central to the EU’s purpose and identity or tie it in with stated EU policy priorities and commitments (Princen 2011: 933). In the “small step” strategy, in contrast, interest groups stress the “apparently technical aspects of an issue and gradually build up support for policies” (Princen 2011: 933).

**Fig. 1:**
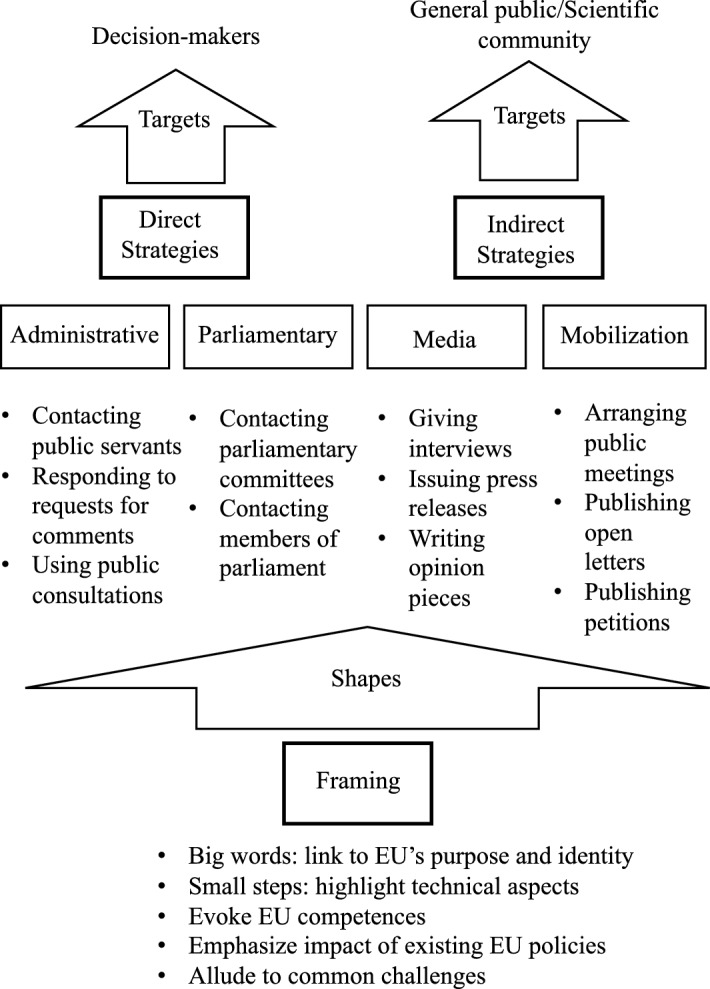
Overview of lobbying strategies and their interrelationship; own illustration

To credibly demonstrate that the EU is the right venue to deal with an issue, it may prove useful to invoke the EU’s competences in that area, highlight how existing EU policies affect the issue in question, or how EU member states may benefit from tackling an issue in concert (Princen 2011: 937-938). Framing thus emerges as another important strategy for interest groups to bring an issue onto the EU policy agenda. Assuming that how an issue is presented is always key, framing is likely to shape all other interest group strategies, direct and indirect (see Figure 1).

## Data and Methods

For my analysis, I triangulated data from semi-structured expert interviews and gray literature. This approach to data collection lends itself to the study of a relatively novel field like AI research policy where data tends to be limited and harder to access.

The data collection was divided into three steps. First, I conducted an unsystematic web search for CERN for AI. This brought up a range of “advocacy” documents, such as newspaper articles for which proponents of the CERN for AI initiative were interviewed. By analyzing these documents, I familiarized myself with the main actors advocating for CERN for AI, their core arguments for the initiative and the EU policy documents on AI that they refer to (for an overview of analyzed media reports and advocacy documents, see Table 1 in the supplementary material). During this phase of the data collection, I also conducted a few exploratory interviews with computer scientists who indicated that the CERN for AI initiative had created controversy within their community and pointed out that several of their colleagues were not in favor of organizing European AI research within a CERN-like ecosystem. 

Second, using the LexisNexis database and the search term “CERN for AI,” I checked that I had not missed any crucial documents during my unsystematic web search. The systematic search generated few new relevant hits.

**Fig. 2:**
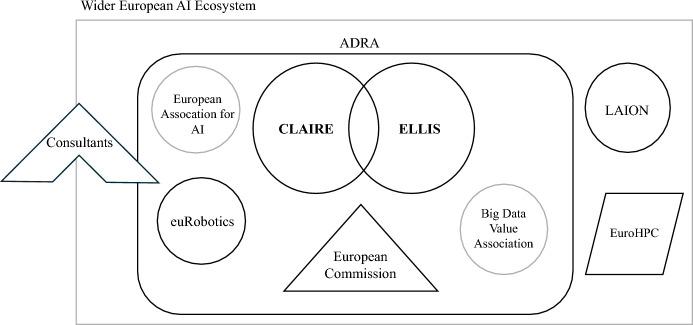
Overview of CERN for AI stakeholders; the CLAIRE and ELLIS circles overlap because some CLAIRE members are also affiliated with ELLIS and vice versa; circles depict the main scientific actors; own illustration

Third, I conducted semi-structured interviews. While my objective was to ensure a balanced perspective through purposeful sampling of all involved stakeholders, proponents of CERN for AI were more responsive than its opponents and policymakers. To counterbalance this self-selection bias, I specifically asked interviewees to recommend and broker contact with opponents of the initiative and with more “neutral” observers like consultants with in-depth knowledge of the wider European AI ecosystem (see Table 2). Between October 2023 and February 2024, I interviewed 19 individuals, most of which were computer scientists affiliated with CLAIRE, the European Laboratory for Learning and Intelligent Systems (ELLIS), the Large-scale Artificial Intelligence Open Network (LAION), or the AI, Data, and Robotics Association (ADRA) and EU policymakers or bureaucrats (see also Figure 2; organizations that interviewees belong to are highlighted in black). In addition, I conducted two informal interviews with a high-ranking EU bureaucrat and a US lawyer that is specialized in AI safety and volunteers for a European AI advocacy group. All interviews were conducted using a semi-structured guideline that slightly varied depending on which stakeholder (policymaker, opponent, proponent or “neutral” observer) I was talking to. In almost all cases, the guideline included questions that asked respondents to reflect on the origins of CERN for AI, the need for such an infrastructure, the main proponents of the initiative, their lobbying strategies and tactics, as well as the future of AI research infrastructure in the EU (see Appendix for an exemplary interview guideline).

**Table 2:** Overview of conducted interviews

Interviewee Code	Source	Main Affiliation	Interview Mode	Interview Length	Date	Language	Transcript
INT01	Sample frame	Confederation of Laboratories for Artificial Intelligence Research in Europe (CLAIRE)	Conducted online via Microsoft Teams	31’	08-Oct-23	ENG	Confidential
INT02	Reference by interviewee from sample frame who declined the interview request	German Research Centre for Artificial Intelligence (DFKI)/CLAIRE	Conducted online via Microsoft Teams	55’	14-Oct-23	GER	Confidential
INT03	Sample frame	CLAIRE	Conducted online via Microsoft Teams	51’	28-Nov-23	ENG	Confidential
Hoos 2023a	Sample frame	CLAIRE	Conducted online via Microsoft Teams	41’	04-Dec-23	GER	Confidential but interviewee agreed to be cited by name
INT05	Sample frame	European Lab for Learning and Intelligent Systems (ELLIS)/Large-scale Artificial Intelligence Open Network (LAION)	Conducted online via Microsoft Teams	42’	05-Dec-23	ENG	Confidential
INT06	Reference by INT02	The AI Data Robotics Association (ADRA)/CLAIRE	Conducted online via Microsoft Teams	25’	18-Dec-23	ENG	Confidential
INT07	Reference by INT02	ADRA/CLAIRE	Conducted online via Microsoft Teams	38’	21-Dec-23	ENG	Confidential
Slusallek 2024	Reference by INT02	DFKI/CLAIRE	Conducted online via Microsoft Teams	51’	17-Jan-24	GER	Confidential but interviewee agreed to be cited by name
INT09	Sample frame	ELLIS	Conducted online via Microsoft Teams	29’	22-Jan-24	ENG	Confidential
INT10	Sample frame	ADRA	Conducted online via Microsoft Teams	45’	22-Jan-24	ENG	Confidential
INT11	Reference by INT06	ELLIS	Conducted online via Microsoft Teams	37’	30-Jan-24	ENG	Confidential
INT12	Sample frame	DG Communications Networks, Content and Technology	Conducted online via Microsoft Teams	22’	31-Jan-24	ENG	Confidential
INT13	Reference by INT11	European High Performance Computing (EuroHPC)	Conducted online via Microsoft Teams	33’	05-Feb-24	ENG	Confidential
Kranzlmüller 2024	Reference by INT12	EuroHPC	Conducted online via Microsoft Teams	28’	05-Feb-24	GER	Confidential but interviewee agreed to be cited by name
INT15	Sample frame	ADRA/euRobotics	Conducted online via Microsoft Teams	54’	08-Feb-24	ENG	Confidential
INT16	Reference by INT13	EuroHPC	Conducted online via Microsoft Teams	26’	08-Feb-24	ENG	Confidential
Marcus 2024	Sample frame	US AI community	Conducted online via Microsoft Teams	10’	13-Feb-24	ENG	Confidential but interviewee agreed to be cited by name
INT18	Reference by INT02	ADRA	Conducted online via Microsoft Teams	36’	16-Feb-24	ENG	Confidential
INT19	Sample frame	DG Research and Innovation	Conducted online via Microsoft Teams with two interviewees present	29’	16-Feb-24	ENG	Confidential
INT20	Sample frame	LAION	Conducted online via Microsoft Teams	20’	20-Dec-23	ENG	Only notes
INT21	Sample frame	DG Research and Innovation	Conducted online via Microsoft Teams	15’	20-Nov-23	ENG	Only notes

Except for the informal interviews, I transcribed all interviews using the software Trint and Microsoft Teams. I analyzed the interview data using MAXQDA and flexible coding (Deterding and Waters 2021). The coding scheme that emerged after several rounds of analysis contains deductive codes that are firmly grounded in the interest group and agenda-setting framework and inductive codes that capture the AI community’s competing visions of how to organize AI research in Europe, the challenges that CERN for AI proponents experienced while promoting the initiative, as well as the history and future prospects for a CERN for AI (see Table 3 below and Table 2 in the supplementary material).

**Table 3:** Codebook

Code	Memo
History of the CERN for AI initiative	This code captures passages that outline when and how the CERN for AI initiative emerged.
Strategies	This code captures the strategies that CLAIRE uses to promote the CERN for AI initiative.
Frames	This code captures what frames CLAIRE uses to promote CERN for AI.
Centralized infrastructure model	This code captures why CLAIRE thinks that a CERN for AI is needed, the arguments for and against organizing AI research in a CERN-like ecosystem and CLAIRE’s vision for a centralized AI research facility.
Decentralized infrastructure model	This code captures what frames scientists use to promote a decentralized organization of AI research in Europe, what advantages they see in having a decentralized AI research network and what they envsion this network to look like.
Challenges	This code captures what challenges CERN for AI proponents faced when advocating for a centralized AI research infrastructure.
Prospects of a CERN for AI	This code captures the different pathways that CERN for AI could take in the future.

The coded material forms the empirical basis for explaining outcome process-tracing, a process-tracing variant whose objective it is to account for a particular outcome (Beach and Pedersen 2016: 309) like, for instance, CERN for AI making it onto the EU policy agenda. According to Beach and Pedersen (2016), “the importance of nonsystematic elements in explaining a particular outcome makes this [process-tracing] variant […] analogous to the historical interpretation of events” (p. 310). Yet explaining outcome process-tracing is distinct from historical research in that it is theory-guided and aims to generalize across a bounded population of cases (Beach and Pedersen 2016: 309-310). Broadly speaking, it is similar to the method of abduction, “where there is a continual and creative juxtaposition of empirical material to theories” (Beach and Pedersen 2016: 311). It is this continual and creative juxtaposition which allows me to provide a rich and “thick” description of the initiation and development of the CERN for AI initiative (Voltolini and Eising 2017: 355).

## “We Need a CERN for AI”

### Issue Initiation: “I Look with Envy at my Peers in High-Energy Physics” (Marcus 2017a)

The proposal to organize AI research in a CERN-like ecosystem first surfaced around 2017. In June 2017, Gary Marcus, then professor of psychology and neural science at New York University, championed the idea—although not yet dubbing it CERN for AI—at the AI for Good Global Summit in Geneva (Marcus 2024). In his keynote speech, he argued that “a bunch of small academic labs may not be enough to get where we need” and that CERN’s way of having thousands of scientists work towards a common cause could prove useful for global AI research, particularly in areas that “industry might otherwise neglect” (Marcus 2017b). Marcus, who is by now one of the most vocal and prominent US critics of the “AI hype,” reiterated these points a month later in a *New York Times* opinion piece. In that piece, he criticizes the common approach of funding AI research through small research laboratories in the academy and large laboratories in industry (Marcus 2017a). According to Marcus, both are inadequate for “grander [AI] projects,” as academic labs lack sufficient scale and funding, while industry labs are often focused on problems that are too narrow in scope. Instead, he suggests that AI research could have greater impact if it were organized in a similarly open and ambitious way as CERN’s Large Hadron Collider (Marcus 2017a).

At about the same time, Philipp Slusallek, professor of computer graphics at Saarland University in Germany, came to similar conclusions—without, however, having heard of Marcus’ proposals (Slusallek 2024). In October 2017, Slusallek attended the 2017 OECD Conference on “AI: Intelligent Machines, Smart Policies,” where he first called for the establishment of a CERN for AI. A physicist by training, Slusallek had always admired the ability of physicists to effectively organize and communicate their research to the world (Slusallek 2024), but had only become familiar with CERN and its ecosystem when he was involved in the European Flagship project “FuturICT” (Bishop et al. 2011). The FuturICT managers had contacted experts at CERN to learn how best to manage a large and long-term research collaboration like a European Flagship (Slusallek 2024). During his interactions with the CERN experts, Slusallek had come to realize that the physics community was successful at getting funding for their experiments because they effectively coordinated their work and were able to agree on a few common objectives (Slusallek 2024). Building on these insights and the discussions held at the OECD conference, Slusallek outlined what in his opinion were the main arguments for a CERN for AI in an article in January 2018. In the article, he criticized that most AI research is “done in isolation, siloed by groups creating their own methods, infrastructures, and datasets” (Slusallek 2018a). Similar to Marcus, he argued that the AI community could overcome this fragmentation by learning from physics, a discipline that is renowned for its “tradition of systematically organizing its key research initiatives around large, strategic projects” (Slusallek 2018a).

### Issue Specification: “A Central Hub for AI in Europe as a Beacon for European Excellence in AI” (CLAIRE 2018b)

In the US, Marcus’ proposal for a CERN-like AI research endeavor initially went unheeded,^2^ but in Europe, the idea caught on, not least because of Slusallek’s advocacy. In late 2017, CERN for AI was first mentioned in an EC report on the “AI policy seminar,” an event that Slusallek attended (Probst et al. 2017: 7). The seminar report states that the idea of “a type of CERN for AI,” broadly understood to be a place “where AI experts could discuss the fundamentals of the technology,” might help “increase transparency in [AI] development and use” (Probst et al. 2017). While it is not specified on whose initiative CERN for AI was brought up during the seminar, it can be assumed that the proposal came from Slusallek, given that he attended the seminar shortly after first advocating for a CERN for AI at the OECD conference in October 2017.

Sometime in 2017, Slusallek also mentioned CERN for AI in an informal exchange with one of his colleagues, Holger Hoos, then professor of machine learning (ML) at Leiden University (Hoos 2023a). Hoos liked the idea. Together with Slusallek and Morten Irgens, one of his long-time colleagues, Hoos co-founded CLAIRE, an initiative aiming to “strengthen European excellence in AI research and innovation” in 2018 (CLAIRE 2023b). Over time, CLAIRE developed into a formal organization with offices and funding. When it was founded, Slusallek, Hoos, and Irgens decided to include the call for a CERN for AI in CLAIRE’s founding document. For Slusallek, there were two underlying motivations for this decision. First, Slusallek and his colleagues perceived a need for more hardware to enable cutting-edge AI research in Europe (Slusallek 2024). Second, and more importantly, CLAIRE’s founding members considered it vital to bring Europe’s top AI researchers together in a central place for them to coordinate their research efforts and to agree on a few top research priorities (Slusallek 2024). While Slusallek, Hoos, and Irgens agreed on these two points, not all of them “were happy with [using] the term CERN for AI [to get this message across],”^3^ mostly because CERN is associated with a different research field and way of working (Slusallek 2024). This may also explain why CLAIRE’s founding document from June 2018 more often refers to a “CLAIRE Hub” than to a CERN for AI.

In CLAIRE’s founding document, the CLAIRE Hub is described as a central node meant to support the network of laboratories that promotes CLAIRE’s mission by providing cutting-edge infrastructure for excellent scientific personnel that are seconded to the hub for limited periods of time (CLAIRE 2018b: 4). As the main authors of the founding document, Slusallek, Hoos, and Irgens clarify that the hub would be modelled on CERN’s “extremely successful” model, meaning a combination of distributed research efforts and “a central facility” (CLAIRE 2018b: 4). They also argue that the hub should be a key element of a broader European AI strategy and that it could be a means to address Europe’s lagging investment and competitiveness in AI research and innovation, thus tapping into the “competition” frame that dominates the global AI discourse (Schiff 2024: 5). Slusallek, Hoos, and Irgens also assert that the CLAIRE hub would help “bring together the […] fragmented AI research activities and expertise in Europe” (CLAIRE 2018b: 4). In September 2018, during a presentation held at a CLAIRE symposium in Brussels, CLAIRE founding member Slusallek further specified that their network should “think for the community at large” and ensure “that all of Europe benefits,” including when it comes to CERN for AI (Slusallek 2018b: 3-6). For CLAIRE, this implied promoting research “across the full spectrum of AI” and not just in ML (CLAIRE 2018b: 2). To raise awareness of their vision among politicians, Slusallek, Hoos, and Irgens made sure that high-ranking EU officials attended events that were of strategic importance to CLAIRE as early as 2018 (see: CLAIRE 2018a), thus engaging in administrative lobbying. In parallel, they began to use media strategies to raise wider awareness of the need to invest into AI research and innovation. For example, in September 2018, CLAIRE issued press releases in response to a prominent special issue on AI in The Economist (CLAIRE 2018c).

In 2018, CLAIRE’s vision partially clashed with that of a group of leading ML researchers. The latter had called for the establishment of a European Laboratory for Learning and Intelligent Systems (ELLIS) in an open letter in April 2018. In the letter, they argued that ELLIS should be modeled on the networked structure of the European Molecular Biology Laboratory (EMBL), should exclusively focus on ML—a technique which at that time they considered to be “at the heart of [the] technological and societal AI revolution”—and should be strictly excellence-driven (ELLIS 2018). Compared to CLAIRE’s broad approach to AI research governance, proponents of the ELLIS network hence advocated for a narrower and more focused organization of European AI research. Despite what one interviewee called a “friendly competition” (INT11) between ELLIS and CLAIRE, both used similar arguments to push for their respective visions of European AI research. For example, similar to CLAIRE, the signatories of the 2018 ELLIS open letter argue that Europe needs to increase its investments in AI research and innovation to keep up with its competitors, big private AI laboratories in the US and China (ELLIS 2018). Both CLAIRE and ELLIS moreover agree that this will require major investments. Yet, while ELLIS underlines that such investments can come from both public and private sources, CLAIRE emphasizes that a CERN for AI should be publicly funded, ideally by the European Commission.

Around 2018, the EC also set up an AI High-Level Expert Group (AI HLEG) with the task of drafting AI ethics guidelines (European Commission 2018: 8). Several prominent CLAIRE members, including Philipp Slusallek, were part of the group. As two interviewees confirmed (INT07; INT03), Slusallek brought CERN for AI up several times during the deliberations within the AI HLEG. Yet, as the group could not reach consensus on the topic, it decided to not include the proposal in any of the AI HLEG reports that appeared throughout 2019. According to two interviewed AI HLEG members, some of their fellow colleagues opposed the idea of a CERN for AI because they expected it to be impossible for politicians to agree on a site for the facility (INT07) and did not see a need for a central research facility in a field like AI (INT03).

The EC White Paper on AI, which was published in February 2020, however, called for something that bears resemblance to a CERN for AI, namely “*a* lighthouse center of research, innovation, and expertise that would coordinate [AI] efforts and be a world reference of excellence in AI” (European Commission 2020: 6; emphasis added). This mention of an AI lighthouse is surprising because according to several interviewed EC officials, the lengthy public consultations running up to the AI White Paper had clearly shown that a majority in the AI community did not favor a central AI hub (INT19), but instead supported a federal, decentralized network of AI excellence centers (INT12). These within-community disagreements may explain why in the AI White Paper the EC also states its intention to “facilitate the creation of excellence and testing centers” (European Commission 2020: 6), which it had already announced in its 2018 Coordinated Plan on AI (European Commission 2018: 4).

To CLAIRE, the EC’s mention of *an* AI lighthouse indicated that their past administrative lobbying had fallen on fertile ground (Slusallek 2024). By then, CLAIRE had become an transnational non-profit organization with headquarters in The Hague and three offices distributed across Europe (Personal Communication Kodde 2024b). This institutionalization process facilitated a coordinated response to relevant AI developments at the EU level. In response to the EC’s White Paper on AI, for instance, CLAIRE promptly issued a press release in which it welcomed the EU’s call for an AI lighthouse, thus engaging in indirect lobbying via the media. CLAIRE’s press release stressed that a central AI research hub is needed for Europe to keep up with global AI research and to counter the influence of private AI laboratories from overseas (CLAIRE 2020: 7). To underline the significance of a central AI research hub for the public good, CLAIRE used a “big word” framing strategy and argued that the development of AI resembles the sequencing of the human genome in the 1990s in that both undertakings have such far-reaching societal consequences that they cannot be left to industry alone (CLAIRE 2020: 7). Taking up a key point from the AI HLEG’s guidelines on AI ethics, namely that AI should align with “the very DNA of the EU” (European Commission 2019), CLAIRE moreover underlined that a European AI lighthouse could help to ensure “that European AI systems, products, and services […] comply with European norms and values” (CLAIRE 2020: 4). In this way, CLAIRE appealed to the EU’s identity as a “normative power” (Manners 2002).

The EC finally launched the AI excellence centers mentioned in its Coordinated Plan and White Paper in late 2020 (see: AI on Demand 2023), with CLAIRE-affiliated researchers managing some and ELLIS-affiliated scientists coordinating others. The European AI lighthouse center for research, however, did not materialize.

### Issue Entrance onto the (Informal) Agenda

In spring 2021, during the deliberations for the European AI Act, the CERN for AI initiative regained momentum and was eventually taken up by members of the European Parliament (MEPs) (Kelly 2021).

At the time, the Directorate-General for Communications Networks, Content and Technology (DG CONNECT), responsible for ensuring “European leadership and independence in critical digital technologies” like AI (European Commission 2024), unveiled its AI approach, which coupled regulation and investment (Kelly 2021). As part of its push for AI investment, the Director-General of DG CONNECT, Khalil Rouhana, announced that the EU was planning to pour money into *several* “lighthouses of AI research” (cited in: Kelly 2021), indicating that the EC was in favor of a networked approach to organizing European AI research. Several MEPs criticized this approach and called for the establishment of a central AI facility, arguing that “more could be achieved by concentrating money” (Kelly 2021). According to an interviewed critic of the CERN for AI initiative, it was unsurprising that some MEPs backed a central European AI facility. This interviewee explained that CLAIRE founding member Holger Hoos had reached out to several MEPs on behalf of CLAIRE to gain their support for CERN for AI (INT11), which aligns with a parliamentary lobbying strategy.

To an interviewed CLAIRE member and proponent of CERN for AI, the EC’s plan to establish a network of AI lighthouses made little sense:

“When we [CLAIRE] talk about it [CERN for AI] it is a physical place. But then this was turned into a network, which to me is an oxymoron. This distributed lighthouse just does not make sense whatsoever. I mean, having a distributed network makes sense. Having one lighthouse makes sense. But a distributed lighthouse does not make sense. It is either or.” (INT07)

According to Slusallek and an interviewed consultant, the establishment of several AI lighthouses moreover risked leading to a further fragmentation of the AI ecosystem in the EU (Slusallek 2024) by “creat[ing] yet another [regional] layer” on top of the already existing layer of decentralized national AI initiatives (INT10).

CLAIRE’s “friendly competitor” ELLIS, in contrast, supported the lighthouse concept, which—considering that ELLIS had called for a European AI ecosystem that resembles the networked structure of EMBL from the very beginning—seems intuitive. An ELLIS member put it like this:

“The lighthouse concept […] is very good because they [the lighthouses] emphasize this attractiveness and visibility […]. Top researchers understand that top research is not everything that happens in society. The lighthouses are not everything that happens, [they] just guide the ships to the harbor. And then there is the harbor and the city and everything that is important happens in the surrounding society.” (INT11)

### Failed Issue Expansion and External Shock: “The ChatGPT-Sputnik Moment” (Hoos 2023a)

In addition to establishing a set of AI excellence centers in 2020, the EC also launched an AI-focused public-private partnership (PPP), ADRA, in 2021. The EC had already announced the initiation of this PPP in its 2018 Coordinated Plan on AI, hoping that it would unite “the [European] AI, data, and robotics community,” as an interviewed ADRA member indicated (INT06). Together with the European Association for AI, CLAIRE and ELLIS represent the AI community in ADRA. For the latter two organizations, the PPP is an important science–policy interface through which they seek to shape AI research and innovation policy. Seen from a mobilization logic of lobbying, ADRA is also a forum that CLAIRE has used as a means to expand the circle of CERN for AI supporters beyond “the [already] convinced” (Cobb et al. 1976). Its advocacy within ADRA has produced mixed results, however.

There are three main reasons for this. First, as an ADRA member and an interviewed consultant commented, within the PPP EC officials tend to neglect what is not already policy, which makes it difficult to push new issues (INT10; INT15). Second, while there is some overlap in membership and objectives between CLAIRE and ELLIS, several prominent ELLIS members in ADRA oppose the CERN for AI initiative. They see no need for a centralized research infrastructure in AI, fear that a central AI facility would isolate “AI research from local industry, research communities, and citizens,” and think that it would absorb large parts of EU funding, but might not necessarily attract promising young researchers (ELLIS 2022). Third, two interviewed ADRA members underlined that it is often hard to reach consensus among the three different communities of ADRA and for them to speak with one voice, as each community is still relatively young and hence preoccupied with its own pet projects and community-building (INT10; INT15).

In November 2022, the communities within ADRA, as well as tech-minded policymakers within the EU, were blindsided by the launch of ChatGPT, OpenAI’s chatbot. An ADRA member summarized the EC’s reaction to ChatGPT as follows:

“The Commission at the moment is a bit like a rabbit in the headlights with respect to the American large language models [LLMs]. It does not know what to do.” (INT15)

Another ADRA member reported that when prominent AI researchers inquired why the EC had not anticipated the importance of LLMs for AI research and innovation when setting up the Horizon program, officials responded that it would have been the AI research community’s duty to inform them about this new trend (INT02). European AI researchers, however, were equally surprised and alarmed by the capabilities and power of the US LLMs (Hoos 2023a). For them, the unexpected release of ChatGPT marked an external shock of almost historic proportions that once more and unequivocally demonstrated the dominance of private actors in leading the field of AI research and innovation (Hoos 2023a).

### Enhanced Issue Specification: “Boost it or Lose it” 

In response to the ChatGPT shock, CLAIRE and other professional science associations increased their advocacy for greater investments into AI research. In spring 2023, LAION, a non-profit organization that seeks to democratize ML research (LAION n.i. 2024), initiated a petition that called for the establishment of a CERN for Open Source Large-scale AI Research (LAION 2023). According to an interviewed LAION member, LAION did not coordinate this initiative with CLAIRE because LAION believes that the AI community should focus exclusively on foundational models, a “scientific transformation” in AI research that in LAION’s view CLAIRE has not yet fully embraced (INT05). With respect to the importance of foundational models for AI research, LAION thus has more in common with ELLIS, as ELLIS started to shift its research focus onto LLMs and foundation models after the release of ChatGPT.

Within CLAIRE, Holger Hoos emerged as a spokesperson for the CERN for AI initiative. According to an interviewed ELLIS member, this made sense because Hoos was known as an eloquent and charismatic personality in the AI community (INT11) and, at that point, had recently been awarded a prestigious Humboldt chair. Throughout 2022 and 2023, Hoos used the visibility attached to being awarded such a chair to promote CERN for AI by giving countless interviews to major newspapers on the state of AI research in Europe and the CERN for AI initiative (e.g. Boldt 2022; Hoos 2023b; Hoos and Irgens 2023). CLAIRE likewise issued two statements that touched on CERN for AI in June and November 2023 (CLAIRE 2023a; CLAIRE and euRobotics 2023). Across all these documents, CERN for AI is largely framed as:

1. A continuation of European success stories in large-scale science and industrial projects,
2. A means to secure European (technical) sovereignty, and
3. A way to develop AI that aligns with European core norms and values.

With respect to the first frame, CLAIRE and Holger Hoos on behalf of CLAIRE often argue that there are several examples of successful, publicly funded European large scientific and industrial projects that demonstrate the merits of investing into a high risk, high gain undertaking like a CERN for AI. Mentioning CERN for AI alongside internationally renowned European large-scale science and industrial projects like CERN, the European Space Agency (ESA), and Airbus has two implications. First, this shows that the EU has a track record of successfully managing megaprojects that rival US initiatives in the same field that it can build on when establishing a CERN for AI. Second, it creates the impression that, like ESA and Airbus, CERN for AI will be a success.

To underline the urgency of investing into a CERN for AI, its proponents moreover tap into the discourse on European technical sovereignty which “pervades the EC’s AI strategy documents” (Mügge 2024: 2). In relation to AI, this sovereignty discourse underlines the need to control the development and deployment of AI technologies, of computing capacity, and data storage. It also emphasizes the importance of having access to human resources and proprietary knowledge to build AI applications and training data (Mügge 2024: 1-2). Typical for a competition-oriented version of “AI sovereignty” are statements which highlight the winner-takes-all dynamic of the field and the high cost of remaining inactive at a time when AI development is at a crossroads (Mügge 2024: 10). In line with this, CLAIRE’s statement on “The Future of AI in Europe” warns:

“Europe is rapidly becoming dependent on AI technologies developed and controlled elsewhere, by a small set of major companies based outside of Europe […]. This technological dependence quickly and necessarily leads to economic and geopolitical dependence […].” (CLAIRE 2023a: 1)

CLAIRE goes on to explain that it is high time for the EU to make a large-scale investment into AI research to counter this trend, arguing that regulation alone will not turn the EU into an AI leader (CLAIRE 2023a: 3) and that its strategy of investing into several distributed, medium-sized AI projects has largely failed to create impact (Hoos 2023b).

Closely related to the “sovereignty” frame, CERN for AI is also regularly portrayed as a means to develop AI that aligns with European norms and values. For instance, in a joint press statement on a “Moonshot in AI,” which would include the establishment of a CERN for AI, CLAIRE and euRobotics—a PPP committed to boosting European robotics research, development, and innovation (euRobotics 2024)—contended that the lack of European leadership in AI is particularly worrying in “areas such as healthcare, education, and law enforcement, where European values are markedly different from those of other countries” (CLAIRE and euRobotics 2023: 1). CLAIRE and euRobotics argue that their proposed moonshot would provide “European alternatives to the generative AI systems created outside of Europe” (CLAIRE and euRobotics 2023: 1). In a similar vein, Hoos argues that a CERN for AI is needed to counter private US tech companies whose power in shaping AI is “detrimental to the preservation of our European value system” (cited in: Boldt 2022). CLAIRE and euRobotics moreover maintain that AI models developed within a CERN for AI could “better reflect and respect cultural differences” within Europe (Matthews 2024).

### Stagnation on the Formal Agenda

Despite CLAIRE’s continuous advocacy for a CERN for AI over the past six years, the initiative has not yet made it onto EU policymakers’ formal decision-making agenda. Why not? There are several reasons that offer explanations.

First and foremost, CLAIRE has failed to counter the AI community’s resistance to CERN for AI and thus to expand the issue beyond those actors that are already convinced by it. To this day, several leading European AI researchers oppose the idea of establishing a central AI research facility in addition to the existing European network of AI excellence centers (INT03; INT09; INT11). This within-community division also puts policymakers off because it signals uncertainty (Slusallek 2024). In line with this, Hermann et al. (1987: 188) have argued that the ability of CERN proponents to “project an image of unity” was key to securing political support and funding for CERN.

Officially, critics of CERN for AI maintain that there is no need to create a central AI research hub because computing infrastructure can be distributed, as can research teams. Beyond such rhetoric, turf battles between different organized scientific interests and CERN for AI’s potential to change the balance of power within the AI community explain the continued resistance towards the initiative. An interviewed consultant, for instance, stated that ELLIS would lose its raison d’être if CLAIRE’s vision of a CERN for AI were ever realized:

“I do not see [ELLIS and CLAIRE working together on CERN for AI]. That would be strange because ELLIS by default claims that they are a network. They would give themselves up by saying we need one CERN because they say: ‘we are that one CERN, we are just decentralized.’” (INT10)

Second, some of the CERN for AI advocates lack experience in navigating the science-policy interface. Slusallek (2024), for instance, stated that he had trouble identifying the right contact points within the EC. Across interviews, CLAIRE members also disagreed as to which lobbying strategies are the most effective. For instance, some interviewed CLAIRE members stressed that media-focused strategies are crucial to push for the CERN for AI initiative (Hoos 2023a; INT15). Another interviewed CLAIRE member emphasized that direct contact with policymakers is much more important (INT02).

Regardless of the tactic chosen, a lack of common language between CLAIRE and EC officials seems to have impeded CERN for AI from gaining traction. An interviewed EC official, for instance, commented that CLAIRE’s advocacy lacked “evidence” and “facts” (INT12), or in other words a solid cost-benefit analysis (INT15). Such analyses are “very difficult to put together,” especially for academics that have little experience with them and, due to their academic commitments, lack the time and resources to get acquainted with them (INT15).

Third, the idea of centralizing a part of the EU’s AI research effort clashes with the fragmented AI (research) policy and funding landscape in Europe. Under the 2019–2024 von der Leyen administration, the topic of AI (research) was split across two DGs, namely DG CONNECT and DG Research and Innovation (RTD). Both feel responsible for AI, but as an interviewed consultant put it, both fundamentally disagree on “what to do with [it] and how,” especially when it comes to AI research infrastructure (INT10). While DG RTD supports a publicly funded research infrastructure for AI in science, an interviewed EU official clarified that DG CONNECT is in favor of a public–private AI initiative:

“I often have the feeling that CLAIRE and ELLIS basically [think]: ‘The Commission should fund this.’ But it cannot always be the Commission. [The money] also has to come from the member states, from private actors. […] It is really important to mobilize everyone, but in particular the private sector.” (INT12)

The European Joint Undertaking on High Performance Computing that provides some infrastructure for AI research further complicates the picture. According to an interviewed European High Performance Computing manager, the complex AI landscape renders it difficult for the different actors within the European AI ecosystem to effectively collaborate (INT13). AI (research) funding, in turn, is as fragmented as the AI (research) policy landscape, with most funding being channeled into several small- to medium-sized projects across Europe (INT07; INT09). According to an interviewed ADRA member, CERN for AI directly counters this decentralized funding strategy:

“[…] this is not how Europe works. Europe is very distributed, and the Commission is always concerned to try and level up the different member states. Picking one country to place a significant facility is always going to produce a problem for the Commission.”(INT15)

This aligns with Hermann et al.’s (1987: 101) conclusion of why CERN succeeded despite initial reservations in the scientific community (p. 101). They argue that no matter how “well-conceived a project may be among scientists themselves, it will not be approved by politicians unless it fits into, or at least does not run counter to, the main lines of policy” (Hermann et al. 1987: 202).

Fourth and finally, CLAIRE’s strategy of framing its proposal for a centralized AI research facility as a *CERN* for AI has proven to be a double-edged sword. On the one hand, several interviewees underlined that the AI community recognizes the power of the CERN “brand” (INT11; INT15). In addition, the interviewees largely agreed that creating a common “roof” under which the AI community can gather is a “sensible idea” (INT15) and may even create a greater community spirit (Kranzlmüller 2024). On the other hand, several interviewees argued that unlike to the massive colliders at CERN, the computing power necessary for AI research does not need to be centralized. This faction maintains that using the CERN label for a large-scale AI research infrastructure is “misleading” (INT11; INT12). Interestingly, in the case of EMBL, proponents used similar framing, going as far as to refer to EMBL as a “Conseil Européen de la Recherche Biologique.” According to Cassata (2024), this analogy between EMBL and CERN “overemphasized the ‘big equipment-intergovernmental laboratory’ connection as the sole institutional framework for organizing scientific and technological collaboration.” A similar argument could be made for the CERN–CERN for AI analogy, although the proponents of CERN for AI have never argued for a fully centralized infrastructure. Instead, they are in favor of combining a central facility with a network of research clusters, preferably those of CLAIRE. In contrast to CERN for AI, the CERN analogy did not backfire in the case of EMBL. This might be because in comparison to CERN for AI proponents, EMBL advocates did a better job of clearly outlining that they did not perceive a decentralized network and a centralized laboratory as “radical alternatives, but as two complementary moments in the process of Europeanization of molecular biology” (Cassata 2024: 9).

### Issue Re-Entrance?

**Fig. 3:**
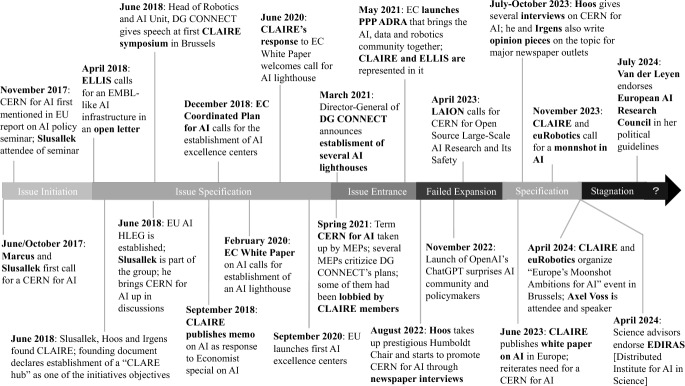
Chronological overview of main CERN for AI events; own illustration

As the previous section has shown, the CERN for AI idea has stagnated on the EU’s informal policy agenda for some time (see Figure 3 for a chronological overview of the main CERN for AI events). In a field like AI, which develops at breakneck speed, and the volatile global political situation of the super election year 2024 (UNDP 2024), however, this can quickly change.

For instance, since the data collection for this study was completed in February 2024, CLAIRE and euRobotics have used the EP elections in June 2024, a period that one interviewee referred to as an important “window of opportunity” (INT15), to intensify their advocacy in Brussels. In doing so, they have not used the CERN for AI label as prominently as before, according to one interviewee because “the CERN for AI phrase got tainted” (INT15) by the criticism it has elicited. Instead, CLAIRE and euRobotics have started to push for a “Moonshot in AI” that would help develop “trustworthy, multicultural generative AI systems for safe physical interaction with the real world” through a European investment of 100 billion euros (CLAIRE and euRobotics 2023: 1). Interestingly, the “moonshot” rhetoric is more prevalent in the US research and development discourse than in the European one, which begs the question of whether it resonates with EU officials.

Moreover, as of spring 2024, the Centre for European Policy Studies, one of Brussels’ most prominent think tanks, endorsed the AI moonshot. This endorsement may indicate that CLAIRE and euRobotics are slowly succeeding in expanding a “rebranded” version of the CERN for AI initiative to a greater audience, which is a prerequisite for an issue to attain systematic agenda standing (in: Cobb and Elder 1972: 110). At the same time, the CERN for AI label seems to have gained popularity with actors other than its original proponents. In April 2024, for instance, it was reported that high-ranking scientific advisors to the EC back a proposal for a “European Distributed Institute for AI in Science” (EDIRAS) that would “take the idea of a CERN for AI forward” (Matthews and Greenacre 2024). CLAIRE opposes EDIRAS as a move “to try and sell something as a CERN for AI which clearly is not” (Personal Communication Kodde 2024a), but it might have a hard time reversing this “misappropriation” of the CERN for AI label, given that an AI in science research facility could appeal to a broader, more diverse (research) community than a research infrastructure that is exclusively used for AI research.

Finally, von der Leyen’s July 2024 proposal to set up “a European AI Research Council where we can pool all of our resources, similar to the approach taken with the CERN” may yet again stir the CERN for AI initiative into a whole new direction (Wulff Wold 2024). The proposal could either indicate that the CERN for AI initiative might soon make it onto the formal policy agenda of the EU or could turn out to be a political move to appease critics of von der Leyen’s AI policy. The fate of CERN for AI thus remains uncertain (for now).

## Discussion and Implications

How does the in-depth case study of the CERN for AI initiative add to our knowledge of STI policymaking in the EU? First and foremost, it sheds light on the role of organized scientific interests in European STI agenda-setting. While the scholarship on STI policy has not neglected to investigate the role of scientists and scientific communities in the policy making process, it has typically depicted them as policy entrepreneurs, knowledge brokers or epistemic communities. So far, scientific communities have not been conceptualized as interest groups, possibly because despite political sociology of science research pointing to the contrary (e.g. Frickel and Moore 2006), the image of the disinterested scientist still prevails. The literature on interest groups, in turn, has largely ignored organized scientific interests, possibly because science policy is often not considered part of the high politics agenda.

Departing from these perspectives, my analysis shows that professional scientific organizations can be just as dexterous as “traditional” interest groups in advancing their interests in STI policy making. Specifically, it uncovers the strategies and tactics through which organized scientific interests shape European STI policy. In the case of CLAIRE’s advocacy for CERN for AI, media- and mobilization-focused strategies prevailed (see Table 4 for an overview of strategies and tactics used). On the one hand, this corroborates previous findings from the interest group scholarship, according to which professional interest groups resemble citizen groups in that both predominantly rely on indirect lobbying strategies (Dür and Mateo 2013: 672). On the other hand, this finding is counter-intuitive, given that the literature on EU interest groups often asserts that media and mobilization strategies are more difficult to employ in the EU context because they target a European public that does not (yet) exist (Kriesi et al. 2007; Mahoney 2008). More recent research on the role of the public in AI policy agenda-setting, however, shows that media-focused strategies can influence what focus policymakers set in AI policy (Schiff 2024). Hence, CLAIRE’s strategy of convincing policymakers, fellow AI researchers, and the broader public that increased AI investments are needed in the EU through interviews, opinion pieces, and public statements may be more effective than the interest group literature suggests. Considering that CLAIRE’s members have countless academic commitments and thus little time to be present in Brussels on a regular basis to directly lobby MEPs and EC officials, media- and mobilization-focused tactics are likely to be a more feasible advocacy approach for them.

**Table 4:** Strategies and tactics used to lobby for CERN for AI; own illustration

Direct strategies		Indirect strategies		
Administrative	Parliamentary	Media	Mobilization	Framing
Participate in consultations (e.g. AI HLEG)	Contact MEPs (Holger Hoos on behalf of CLAIRE)	Give interviews on CERN for AI (e.g. Die Welt, Tagespiegel)	Organize own events (e.g. CLAIRE symposia, Moonshot in AI event in Brussels)	Big words (CERN for AI as a means to create AI that aligns with European values)
Use PPPs (ADRA)		Write opinion pieces about the need for a CERN for AI (e.g. in Science Business)	Convincing ADRA members of CERN for AI’s merit	Portray EU as the right venue to tackle CERN for AI (stressing the EC’s failure to create impact with existing AI research programmes)
		Issue press releases (e.g. in response to EC White Paper on AI, Economist special issue on AI)		

In addition to media and mobilization strategies, the case study also revealed that CLAIRE made use of selected administrative and parliamentary tactics when advocating for CERN for AI. For example, prominent CLAIRE members provided input to public consultations at the EC level and contacted MEPs to convince them about CERN for AI. Actively creating opportunities to interact with policymakers seems to be a common approach among scientists that are advocating for costly research infrastructure. In the case of the African Light Source (AfLS), an initiative that seeks to establish a synchrotron light source on the African continent, for example, project proponents created a “minister forum” where officials and scientists are intended to discuss the prospects of an AfLS (Rüland et al. 2023: 788).

Finally, the analysis has shown that to credibly present the EU as the right venue to deal with CERN for AI, CLAIRE highlighted how the EU’s past regulatory and investment approach to AI failed to create noteworthy impact. By framing CERN for AI as a) a continuation of a long line of successful European big science and industrial projects, b) a means to ensure European (technical) sovereignty, and c) a way to develop AI that aligns with European norms and values, CLAIRE coupled its criticism of the EU’s past approach to AI with a “big word” framing strategy. Proponents of other large-scale infrastructures have engaged in similar framing strategies, as has been shown in section “Stagnation on the Formal Agenda.”

Coming back to the question that opened this section, the case study of the CERN for AI initiative also offers important lessons for computer scientists that are interested in shaping and policymakers that are involved in crafting AI policies. It specifically demonstrates why and where frictions arise between organized scientific interests and policymakers that are trying to bridge the research–policy intersection of AI and to organize a research field of strategic interest. Section “Enhanced Issue Specification: “Boost it or Lose it”,” for instance, showed that some of the CERN for AI proponents felt like they lacked experience in the policy realm, struggled to “speak the language” of policymakers, and did not have the time or the resources to lobby effectively. Research has shown that most academics consider engagement with decision-makers for the purpose of influencing public policy a duty (Jessani et al. 2022), but that they feel ill-prepared to do so. The problem might be more pronounced for computer scientists who, until the “AI hype,” have been presented with fewer opportunities to engage with policymakers due to the technical nature of their research. Computer scientists and organized scientific interests with policy ambitions in the AI realm might therefore want to strengthen their own as well as their members’ soft skills and exposure to the political arena. Alternatively, they could consider collaborating with consultants that have lobbying experience to further their respective policy proposals. Given that an interviewed EU official perceived a lack of willingness or skill on CLAIRE’s and ELLIS’ part to secure private funds for a large-scale AI research infrastructure, such consultants could help organized scientific interests to convince private actors to invest into a private-public research infrastructure.

Section “Enhanced Issue Specification: “Boost it or Lose it”” also demonstrated that the fragmented governance of an emerging research field like that of AI between different DGs, PPPs, and policy initiatives creates inefficiencies and hampers community-building. In the case of AI research policy, these challenges could be tackled if the topic were brought together under the same organizational “roof” within the EC or if coordination between DG RTD and DG CONNECT on AI-related initiatives were improved.

## Conclusion

In this paper, I investigated the role of organized scientific interests in STI policy agenda-setting by tracing how CERN for AI made it onto the informal policy agenda of EU decision-makers. Given that the findings from such a single case are hard to generalize, future studies should explore the extent to which CLAIRE’s strategies and tactics differ from or resemble those of other organized scientific interests like, for example, the AfLS Foundation, a fierce advocate of an AfLS. Such studies could also clarify whether organized scientific interests in other disciplines face similar challenges to those faced by CERN for AI proponents. Moreover, while this study aimed to draw some parallels between the agenda-setting phase of CERN and CERN for AI, additional historical comparisons could systematically examine the conditions under which organized scientific interests succeed in putting a proposal for large-scale research infrastructure onto policymakers’ formal agenda.

From a theoretical point of view, further work is needed to develop the concept of organized scientific interests for STI policy studies. It would specifically be helpful to more clearly delineate the concept from related theoretical constructs, such as that of the policy entrepreneur. Such an exercise has merit because the concept of organized scientific interests could help explain how scientists and their respective communities effectively pursue their interests in an increasingly competitive research and funding environment.

## Supplementary Information

Below is the link to the electronic supplementary material.

Supplementary file1 (DOCX 600 KB)

## Footnotes

1 In late 2024, the Confederation of Laboratories for AI Research in Europe was made aware that the acronym it had been using for the past five years was trademarked. As a result, the confederation changed its acronym from CLAIRE to CAIRNE (pronounced “kern”) (CAIRNE 2024), while keeping its full legal name unchanged. In the following, I use CLAIRE instead of CAIRNE because the article almost exclusively focuses on a period during which the confederation was still known as CLAIRE.

2 Although the US government did underline a more general need for AI research and safety infrastructure in key policy documents, including in its National Artificial Intelligence Research and Development Plan of 2016, 2019 and 2023.

3 All quotes from interviews that were conducted in German were translated by the author.
