# Supplementary material for: “We Need a CERN for AI”: Organized Scientific Interests and Agenda-Setting in European Science, Technology, and Innovation Policy
Source: Minerva. 2025 Mar 17;64(2):407–36. doi: 10.1007/s11024-024-09568-6 (PMC13253650; doi:10.1007/s11024-024-09568-6)
Supplement: Supplementary file 1 — Supplementary file1 (DOCX 600 KB) [file 11024_2024_9568_MOESM1_ESM.docx]

**Appendix**

Table 1: Overview of analyzed documents

Table 2: Coding scheme with exemplary quotes

**Exemplary interview guideline (scientists)**

1. When did the idea of a CERN for AI first come up?
2. Why is a CERN for AI needed? Why are the existing four networks of AI excellence (AI4Media, ELISE, Human-AI-net, TAILOR & VISION) not sufficient?
3. What would a CERN for AI look like? What role would the EU/individual member states play in a CERN for AI?
4. Who advocates for a CERN for AI? To what extent do you cooperate or coordinate your own advocacy with that of other actors? To what extent do your visions of a CERN for AI converge/diverge?
5. Who do you lobby?
6. Do you directly approach EU and/or national policymakers to promote a CERN for AI? If so, how do you establish contact?
7. Do you directly approach EU and/or national bureaucrats to promote a CERN for AI? If so, how do you establish contact?
8. How do you advocate for a CERN for AI?
9. Do you use media to advocate for a CERN for AI? How exactly and which media?
10. Do you mobilize colleagues to advocate for a CERN for AI?
11. Do you use different advocacy strategies at the EU level than you do at the national level?
12. Which strategy do you deem most effective in promoting a CERN for AI? Why?
13. Do you think that your advocacy will eventually be successful?
14. How has your advocacy for a CERN for AI been received by the scientific community/policymakers/media?
